# Integrated metasurfaces on silicon photonics for emission shaping and holographic projection

**DOI:** 10.1515/nanoph-2022-0344

**Published:** 2022-10-20

**Authors:** Ping-Yen Hsieh, Shun-Lin Fang, Yu-Siang Lin, Wen-Hsien Huang, Jia-Min Shieh, Peichen Yu, You-Chia Chang

**Affiliations:** Department of Photonics, College of Electrical and Computer Engineering, National Yang Ming Chiao Tung University, Hsinchu 30010, Taiwan; Taiwan Semiconductor Research Institute, Hsinchu 30078, Taiwan

**Keywords:** holography, metasurfaces, silicon photonics

## Abstract

The emerging applications of silicon photonics in free space, such as LiDARs, free-space optical communications, and quantum photonics, urge versatile emission shaping beyond the capabilities of conventional grating couplers. In these applications, silicon photonic chips deliver free-space emission to detect or manipulate external objects. Light needs to emit from a silicon photonic chip to the free space with specific spatial modes, which produce focusing, collimation, orbital angular momentum, or even holographic projection. A platform that offers versatile shaping of free-space emission, while maintaining the CMOS compatibility and monolithic integration of silicon photonics is in pressing need. Here we demonstrate a platform that integrates metasurfaces monolithically on silicon photonic integrated circuits. The metasurfaces consist of amorphous silicon nanopillars evanescently coupled to silicon waveguides. We demonstrate experimentally diffraction-limited beam focusing with a Strehl ratio of 0.82. The focused spot can be switched between two positions by controlling the excitation direction. We also realize a meta-hologram experimentally that projects an image above the silicon photonic chip. This platform can add a highly versatile interface to the existing silicon photonic ecosystems for precise delivery of free-space emission.

## 1 Introduction

Silicon photonics has recently shown promises in delivering free-space emission to detect or manipulate external objects with multifunctional photonic chips. Notable applications include light detection and ranging (LiDAR) [1–7], free-space optical communication [8, 9], display [10, 11], optogenetics [12, 13], and quantum computation [14, 15]. Silicon photonic platform offers scalability, reconfigurability, compactness, parallelism, and mature fabrication, thanks to the complementary metal-oxide-semiconductor (CMOS) compatibility [16]. Silicon optical phased arrays, for example, have leveraged the scalability to integrate hundreds or thousands of phase shifters to form and steer free-space beams, offering the key functions for LiDAR and free-space optical communication [1, 3, 4]. In optogenetics and trapped-ion-based quantum computation, silicon photonics offers precise light delivery to the neuron or ion positions as well as the scalability to increase the number of addressable neurons and qubits, which is challenging for conventional bulk optics [12–15]. These emerging applications urge the generation of precise and sometimes complicated free-space spatial modes, including beam focusing [14, 17], collimation [18, 19], orbital angular momentum generation [20], and holographic projection [11]. Beam focusing allows optical excitation or sensing exclusively of a small volume in space, which is crucial for addressing qubits in quantum photonics and neurons in optogenetics [14, 21]. Generating a large-aperture collimated beam is the key to extending the ranging distance in LiDARs [18, 19]. Beams carrying different orbital angular momentums can enable spatial-division-multiplexing for free-space optical communication [20].

The emerging free-space applications of silicon photonics urge versatile emission shaping beyond the capabilities of conventional grating couplers and inverse tapers [22, 23]. Although grating couplers can be apodized with a spatially varying period to perform beam focusing [14], generalization to arbitrary shaping is challenging. To address the need for arbitrary free-space beam shaping, metasurfaces have been introduced to silicon photonics. Metasurfaces are planar arrangement of subwavelength-spaced optical scatters, the so-called meta-atoms [24, 25]. Metasurfaces can impose local modulation on the phase, amplitude, polarization, and even dispersion of electromagnetic waves by assigning the geometrical parameters of each scatter [24–28]. While there has been a vast amount of research using metasurfaces as free-space components [29–31], the integration of metasurfaces on photonic integrated circuits has yet been fully explored [2, 17, 32], [33], [34], [35], [36], [37], [38]. In the works by Chang et al. and Yulaev et al. [2, 17], silicon metasurfaces are placed on top of grating couplers to either collimate or focus the grating emission. However, because the metasurface and the photonic integrated circuit are fabricated separately, precise alignment and packaging are required. Monolithic integration of metasurfaces on silicon photonic integrated circuits has been proposed numerically [32, 33, 38] and demonstrated experimentally [34, 35]. Ding et al. demonstrate a metasurface monolithically integrated on a silicon waveguide to create 2D beam focusing [34]. However, Au–Si–Au sandwiched nanobars, the meta-atoms used in Ref. [34, 35], are incompatible with the standard CMOS process and thus cannot be fabricated in foundries. The resonance-based phase shifting mechanism of the Au–Si–Au sandwiched nanobars also limits the precision of wavefront shaping because of the high sensitivity to the meta-atom dimensions.

In this paper, we report a monolithic and CMOS-compatible platform to integrate metasurfaces on silicon waveguides for versatile shaping of free-space emission. We demonstrate experimentally versatile wavefront shaping, including 2D diffraction-limited beam focusing with a Strehl ratio of 0.82 and holographic projection of an image above the chip. We fabricate the meta-atoms of amorphous silicon (a-Si) nanopillars on top of silicon waveguides. The CMOS compatibility of our platform allows monolithic and scalable integration of the metasurfaces with photonic integrated circuits. Simulation shows that the phase shift is introduced by the propagation through the nanopillar without relying on sensitive resonances, which enables more reliable phase control in practice compared to previous works (see Supplementary Material for detailed robustness comparison) [34, 35]. We demonstrate that the meta-atoms evanescently coupled to a waveguide mode can convert the guided mode excitation to the free-space emission with well-determined phase shifts. The demonstrated metasurfaces can integrate with various waveguiding components to form a comprehensive chip-scale optical system for free-space applications.

## 2 Design and simulation

**Figure 1:**
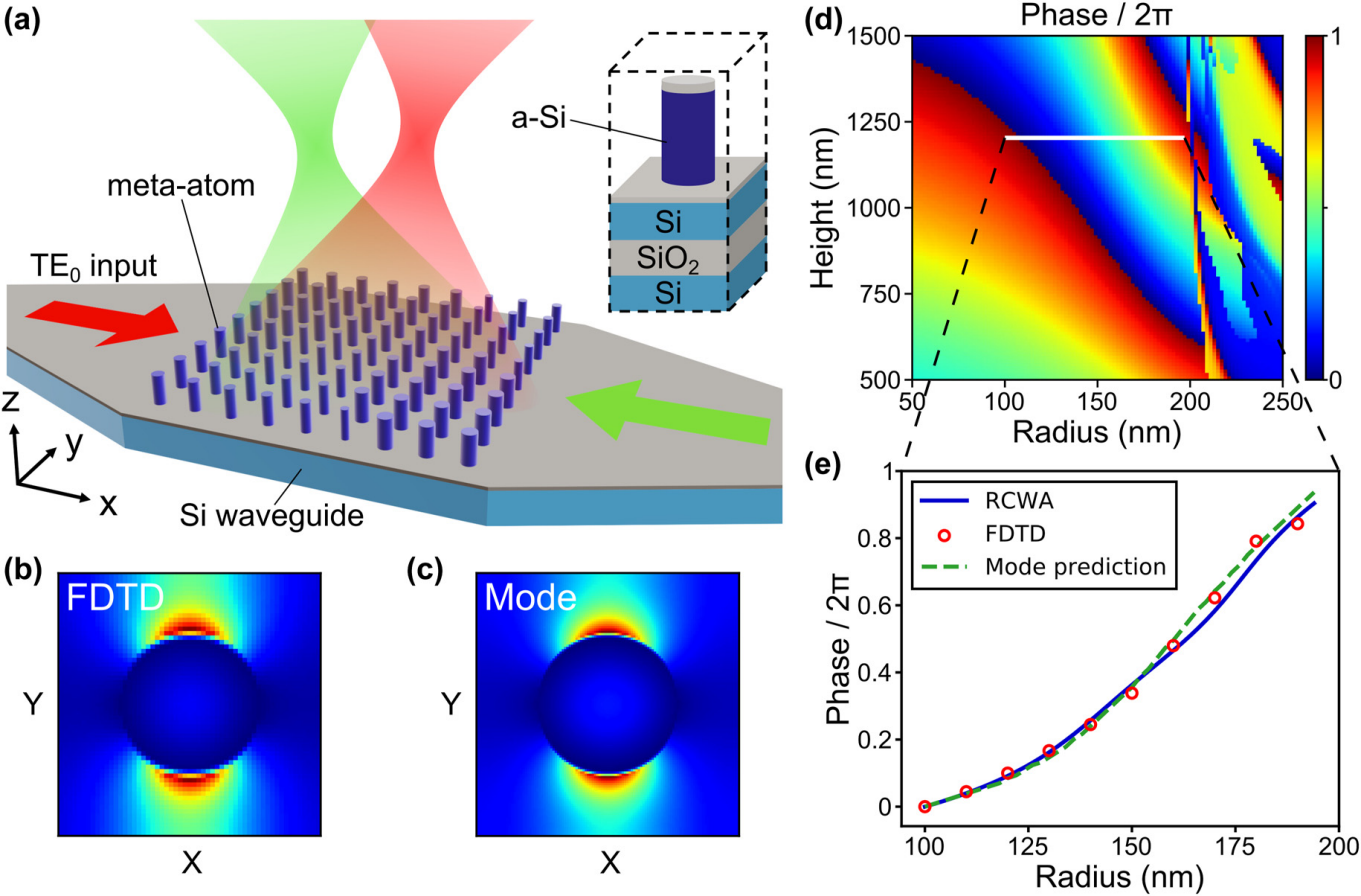
Schematic and simulation of the monolithic platform of metasurfaces on silicon photonics. (a) Schematic of a metasurface integrated on a silicon waveguide for switchable beam focusing. The metasurface consists of a-Si nanopillars in a square lattice. The metasurface is excited by the guided mode and creates focusing emission. The focused spot can be switched between two positions, depending on the excitation direction, as indicated by the green and red colors. Inset: schematic of the meta-atom unit cell (height: 1.2 µm; period: 562 nm). (b) FDTD-simulated intensity distribution near one of the nanopillars in the lattice when excited by the guided mode. The nanopillar radius is 150 nm. (c) Eigenmode of a nanopillar in the lattice (nanopillar radius: 150 nm). (d) RCWA-simulated phase of the emission from the nanopillar array as a function of the nanopillar height and radius. (e) Meta-atom phase library used in the design, where the radius ranges from 100 to 194 nm. We confirm the phase response with three different simulation methods: RCWA (blue solid line), 3D FDTD (red circle), and mode prediction (green dashed line), showing the phase response originates from the propagation phase delay in each nanopillar.

We design metasurfaces monolithically integrated on standard 220 nm-thick silicon photonic waveguides for generating shaped emission, as shown in Figure 1a. The metasurfaces are composed of meta-atoms of 1.2 µm-tall a-Si nanopillars. The operating wavelength is 1550 nm. We arrange these nanopillars in a square lattice with a period Λ of 562 nm. The period is chosen to allow easier vertical emission while avoiding the back reflection from the second-order diffraction (see Supplementary Material). These a-Si nanopillars couple to the TE_0_ mode of the silicon photonic waveguide evanescently, perturbing the guided mode to create light emission to the free space. To ensure perturbative coupling strength, we introduce an 80 nm SiO_2_ interlayer between the waveguide and the a-Si nanopillars. We perform 3D finite-difference time-domain (FDTD, Lumerical Inc.) simulation to study the intensity distribution in one of the evanescent-coupled meta-atoms in the lattice, as shown in Figure 1b. The intensity distribution is consistent with the eigenmode of a nanopillar in the lattice shown in Figure 1c. This indicates that an evanescent-coupled meta-atom in a lattice can be viewed as a small truncated cylindrical waveguide. Each nanopillar can introduce a phase shift to the emission, controlled by its effective refractive index $n_{\text{eff}}^{\text{pillar}}$ and thus by the nanopillar radius. This mechanism has been used in the literature to realize free-space polarization-independent metalenses [39]. We can thus engineer the emission from the waveguide and realize arbitrary phase profiles simply by varying the nanopillar radii at different positions. In contrast to conventional grating couplers in silicon photonics [21, 22], our metasurfaces allow phase assignment down to the unit cell level, enabling versatile wavefront shaping and holographic projection. We establish a library of meta-atom design parameters by performing simulation with the rigorous coupled-wave analysis (RCWA) [40], as shown in Figure 1d and e. With this library, we can look up the required geometrical design parameter at each position. RCWA allows a more efficient search of design parameters than the more rigorous but computation-intensive 3D FDTD. To simulate the evanescently-coupled nanopillar array in RCWA, we excite the nanopillars with a total internal reflected (TIR) plane wave source. The incident angle of the plane wave *θ*_i_ is chosen to be $\text{s}\text{i}{\text{n}}^{-1}\left(n_{\text{eff}}^{\text{slab}}/n_{\text{S}\text{i}}\right)$ to produce the same evanescent field of the TE_0_ slab waveguide mode, where $n_{\text{eff}}^{\text{slab}}$ is the effective refractive index (see Supplementary Material). Figure 1d shows the phase of the emission from the nanopillar array as a function of the nanopillar height and radius. Considering the fabrication capabilities in our facility, we choose a height of 1.2 µm and radii ranging from 100 to 194 nm. As shown in Figure 1e, this library provides phase coverage of 1.9*π*, which is sufficient to produce almost any emission phase profile. We compare the emitting phase library obtained by RCWA with the more rigorous 3D FDTD simulation and obtain a good agreement. These results also match well with the phase response predicted by the eigenmode of the nanopillar (see Supplementary Material). The agreement confirms the physical origin of the phase response: each evanescently-coupled nanopillar can be treated as a truncated cylindrical waveguide to produce a propagation phase delay determined by its radius.

**Figure 2:**
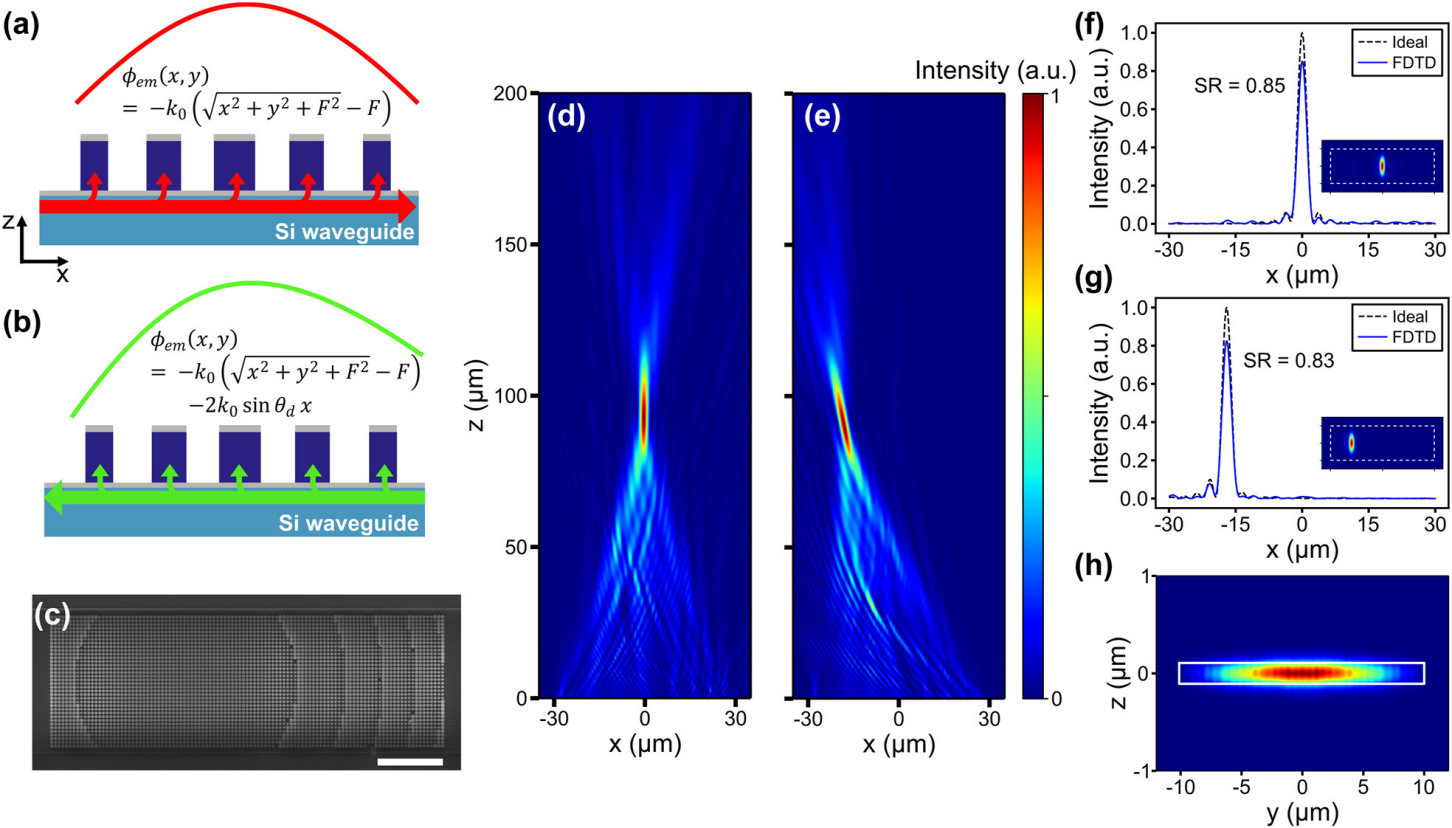
Design methodology and simulation of switchable beam focusing. (a, b) Illustration of the emission phase profile when the metasurface is excited by the +*x* (−*x*) propagating guided mode. (c) SEM image of the fabricated metasurface, showing the metasurface pattern for switchable beam focusing. Scale bar: 10 μm. (d, e) FDTD-simulated focusing intensity distribution on the *xz* plane when the metasurface is excited by the +*x* (panel d) and −*x* (panel e) propagating guided modes. The emission angle is tilted by −11° in panel e, showing a switchable operation. (f, g) Cross-sections of the FDTD-simulated intensity distributions at the focal plane when the metasurface is excited by the +*x* (panel f) and −*x* (panel g) propagating guided modes. For comparison, we also plot the ideal intensity distributions produced by the uniform emission from a rectangular aperture with ideal hyperbolic phase profiles. The simulated Strehl ratios are 0.85 and 0.83 in panels d and e, respectively. The intensity is normalized to the integrated power. Insets: 2D intensity distributions on the focal plane, where the white dashed line indicates an area of 60 μm × 20 μm. SR: Strehl ratio. (h) Simulated intensity distribution in the 22 µm × 220 nm silicon waveguide after the light passes through the metasurface-coupled region, in which 99% of the power remains in the TE_0_ mode.

With the phase library, we design a metasurface to produce focusing emission from a waveguide, where the focused spot position can be switched by controlling the excitation direction, as illustrated in Figure 1a. When the excitation guided mode propagates in the +*x* direction, it inherits the accumulating propagation phase *βx*, where *β* is the propagation constant (Figure 2a). The phase profile *ϕ*_em_(*x*, *y*) emitted to the free space can be expressed as *ϕ*_em_(*x*, *y*) = *ϕ*_ms_(*x*, *y*) + *βx*, where *ϕ*_ms_(*x*, *y*) is the abrupt phase shift created by the metasurface. To produce a focused spot with a focal length *F* normal to the metasurface, we choose the metasurface phase profile to be

(1) $$\phi_{ms}\left(x,y\right)=-k_{0}\left(\sqrt{x^{2}+y^{2}+F^{2}}-F\right)-\beta x,$$

where the last term cancels with the propagating phase *βx* to generate the ideal hyperbolic phase profile for the focusing emission [24]. Here we design a 60 μm × 20 μm metasurface with *F* = 100 μm. This metasurface phase profile *ϕ*_ms_(*x*, *y*) can be realized by looking up the proper meta-atom radii from the library. The corresponding metasurface pattern is shown by the scanning electron microscope (SEM) image of the fabricated metasurface in Figure 2c. The pattern is asymmetric in the *x* direction due to the last term in Equation (1). The same metasurface produces a different emission phase profile when it is excited by the −*x* propagating guided mode, as depicted in Figure 2b. The emission phase profile becomes $\phi_{em}\left(x,y\right)=\phi_{ms}\left(x,y\right)-\beta x$ Using the grating equation *β* = *k*_0_sin*θ*_d_ + 2*π*/Λ we can rewrite the emission phase profile as

(2) $$\phi_{em}\left(x,y\right)=-k_{0}\left(\sqrt{x^{2}+y^{2}+F^{2}}-F\right)-2k_{0}\sin\theta_{d}x,$$

where *θ*_d_ is the emission angle for a grating. The last term creates a tilt to the wavefront. Therefore, given Λ = 562 nm and *β* = 2.8535 *k*_0_ for the TE_0_ mode, when we send the excitation guided mode in the −*x* direction, the focusing emission from the metasurface is tilted by −11°.

We perform 3D FDTD simulation to verify the switchable beam focusing from the metasurface and show diffraction-limited focusing quality. Figure 2d shows the emission intensity distribution on the *xz* plane when the metasurface is excited by the +*x* propagating guided mode. We observe a tightly focused spot at the height of 94 µm with a full width at half maximum (FWHM) spot size of 2.3 µm (*x* direction) by 8.2 µm (*y* direction). When we excite the metasurface with the −*x* propagating guided mode, as shown in Figure 2e, the emission is tilted by −11°, which is consistent with Equation (2). The focused spot has an FWHM spot size of 2.4 µm (*x* direction) by 8.1 µm (*y* direction) at the height of 90 µm. The focusing efficiency for the +*x* propagating excitation case is 11% (see Supplementary Material for the definition), while 65% of the input power passes through the metasurface-coupled region and remains in the waveguide. The focusing efficiency for the −*x* propagating excitation case is 14%. In this case, 56% of the input power remains in the waveguide after passing through the metasurface region. Detailed analysis of the energy flow, including upward and downward emission, transmission, and back reflection, are provided in the Supplementary Material. The focusing efficiency can be further improved by increasing the length of the metasurface in the *x* direction to extract all the waveguide power. Figure 2f and g show the cross-sections of the simulated intensity distributions at the focal plane along the *x* direction for the two excitation directions. We compare the simulated intensity distributions from the metasurface with the ideal intensity distributions produced by the uniform emission from a rectangular aperture of 60 μm × 20 μm with ideal hyperbolic phase profiles. We calculate the Strehl ratios from the comparison with the ideal intensity distributions (see Supplementary Material). The simulated Strehl ratios are 0.85 and 0.83 for the +*x* and −*x* propagating excitation cases, respectively, indicating diffraction-limited performances. We analyze the mode purity of the light remaining in the waveguide after passing through the metasurface-coupled region. The intensity distribution obtained by 3D FDTD is shown in Figure 2h. Modal expansion shows that a fraction of 99% remains in the TE_0_ mode, indicating that the perturbation of waveguide-metasurface coupling does not excite any significant higher-order modes.

## 3 Fabrication and measurement

**Figure 3:**
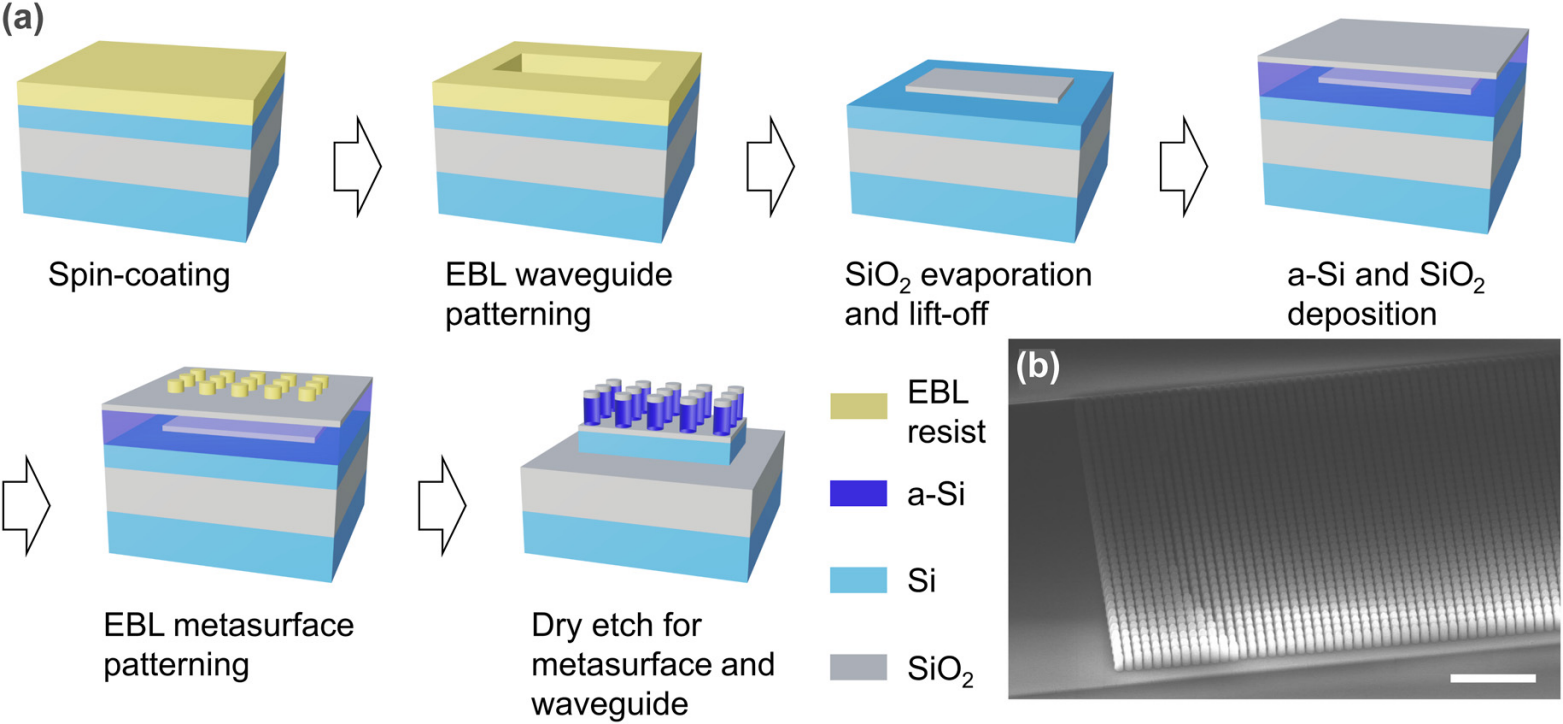
Fabrication processes and image of the devices. (a) Fabrication processes of the metasurface-on-waveguide structure using EBL and a single etching step. (b) Tilted SEM image of the metasurface monolithically integrated on a silicon waveguide. Scale bar: 5 μm.

We develop a process to fabricate the metasurface and the silicon waveguides using electron beam lithography (EBL) and a single plasma etching step, as shown in Figure 3a. First, we spin-coat electron-beam photoresist (ZEP 520A) on a silicon-on-insulator (SOI) substrate with a 3 µm buried oxide layer and a 220 nm top silicon layer. This is followed by the waveguide patterning using EBL. Electron beam evaporation is used to deposit 80 nm SiO_2_, followed by the lift-off process to make the SiO_2_ etch mask for the waveguides. After the waveguides are defined, we deposit 1.2 µm a-Si and 150 nm SiO_2_ by plasma-enhanced chemical vapor deposition (PECVD). We spin-coat electron-beam photoresist (ma-N 2403) and pattern the metasurface structure with precise alignment. We use inductively-coupled plasma reactive-ion etching (ICP-RIE) to transfer the metasurface pattern to the SiO_2_ etch mask using C_4_F_6_/Ar/O_2_ based gases. After that, a single ICP-RIE etching step using HBr/Cl_2_ based gases creates the metasurface and the waveguides together. Figure 3b shows the SEM image of the fabricated device. We fabricate the whole chip, including photonic integrated circuits and integrated metasurfaces, using the described process. Detailed characterization of the waveguide propagation loss is given in the Supplementary Material.

**Figure 4:**
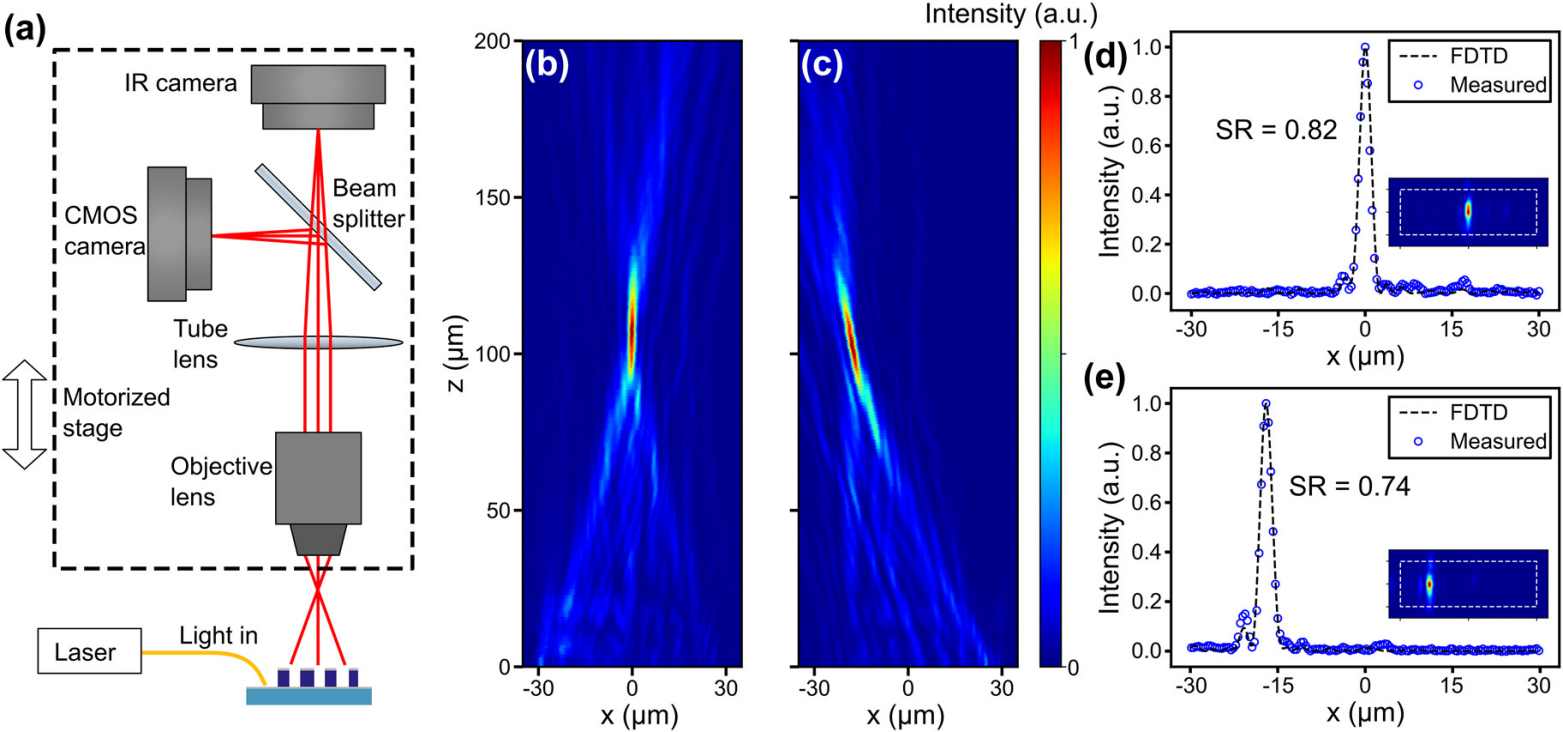
Measurement of the integrated metasurface for generating a switchable focusing emission profile. (a) Schematic of the measurement setup. (b, c) Measured intensity distributions on the *xz* plane when the metasurface is excited by the +*x* (panel b) and −*x* (panel c) propagating guided modes. The emission angle is tilted by −10° in panel c, showing a switchable operation. (d, e) Cross-sections of the measured and FDTD-simulated intensity distributions at the focal plane when the metasurface is excited by the +*x* (panel d) and −*x* (panel e) propagating guided modes. The peak intensity is normalized to 1. The measured Strehl ratios are 0.82 and 0.74 in panels d and e, respectively. Insets: 2D intensity distributions on the focal plane, where the white dashed line indicates an area of 60 μm × 20 μm. SR: Strehl ratio.

We measure the emission from the monolithically-integrated metasurface and demonstrate switchable diffraction-limited beam focusing with a Strehl ratio of 0.82. The measurement setup is shown in Figure 4a. The light source is a tunable narrow-linewidth laser (Toptica CTL1550) operating at the wavelength of 1550 nm with a linewidth <10 kHz. We couple the light from a cleaved single-mode fiber to the silicon photonic integrated circuit with a grating coupler. Light is routed by the silicon waveguides to the regions with the integrated metasurface, where light emits to the free space. The emission from the metasurface is captured by a home-built infinite-conjugate microscope with a 50X NA 0.65 objective (Mitutoyo M Plan Apo NIR) and an infrared camera (Xenics Bobcat 320). We translate the microscope with a motorized stage to image along different heights to map the 3D intensity distribution of the emission. The use of a high NA objective allows a short depth of focus of 1.83 µm to section the intensity distribution plane by plane (see Supplementary Material). Figure 4b and c show the measured intensity distributions on the *xz* plane when the metasurface is excited in the two different directions. When guided mode excitation is along the +*x* direction, a focused spot is formed vertically at *z* = 109 µm. As we switch to the opposite excitation direction, the focusing profile is titled by −10° and forms a spot at *z* = 102 µm. The slightly larger focal lengths and different tilt angle compared to the simulations are attributed to the meta-atom dimension errors in the fabrication. The cross-sections of the measured intensity distributions at the focal plane along the *x* direction are shown in Figure 4d and e, which agree with the FDTD simulations. The insets show the focused spot on the *xy* plane. In Figure 4d, the measured FWHM spot size is 2.1 μm (*x* direction) by 6.5 μm (*y* direction). The Strehl ratio is 0.82, indicating a diffraction-limited performance. In Figure 4e, the measured FWHM spot size is 2.2 μm (*x* direction) by 5.8 μm (*y* direction), and the Strehl ratio is 0.74. In the *y* direction, the measured FWHM spot sizes are smaller than the simulated values, which we attribute to the slightly distorted profiles along this direction (see Supplementary Material).

**Figure 5:**
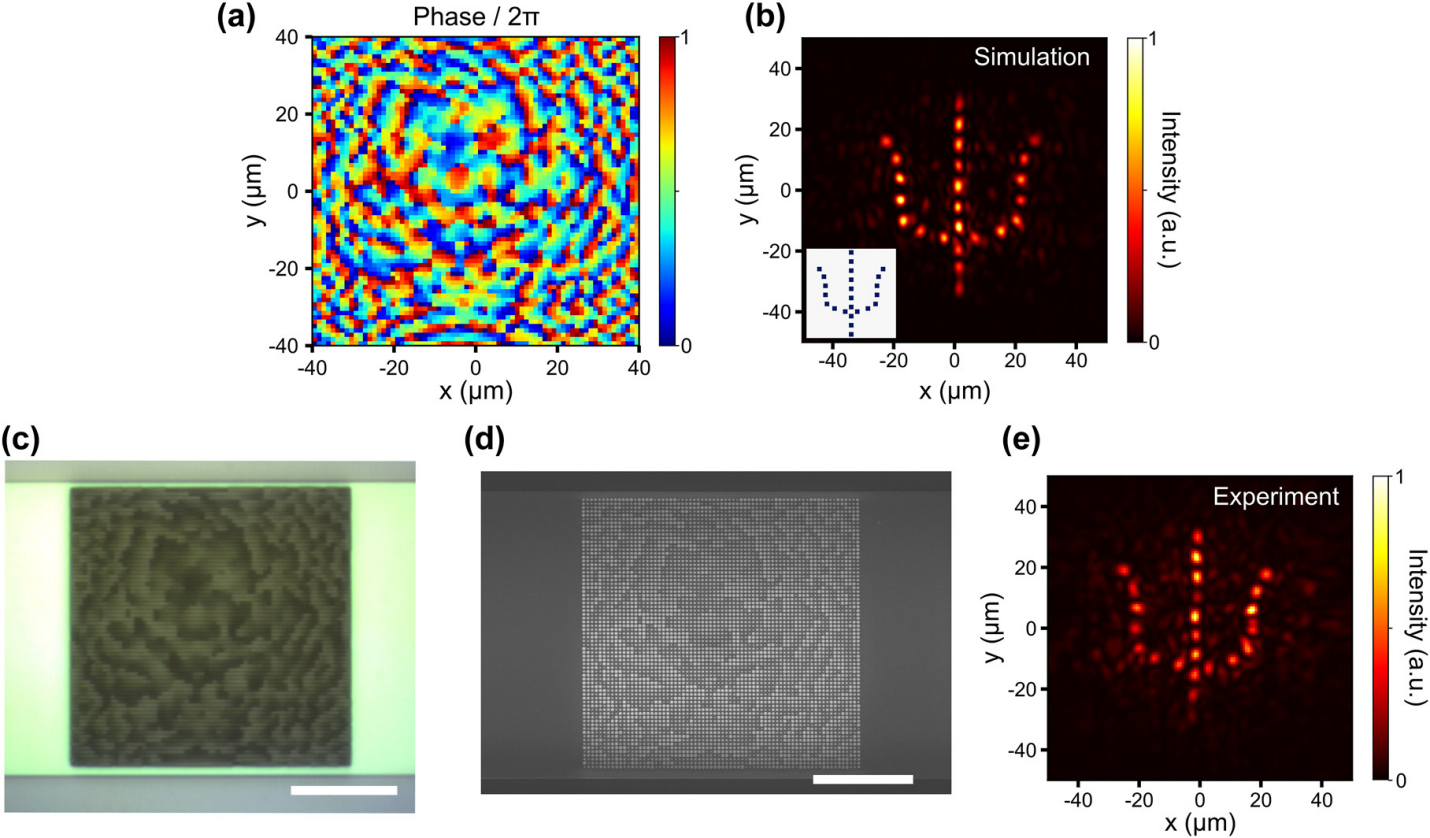
Simulation and experimental demonstration of an integrated meta-hologram. (a) Phase profile obtained by the Gerchberg–Saxton algorithm for projecting the letter “ψ” at 20 µm above the metasurface. (b) Simulated intensity distribution at 20 µm above the metasurface obtained by FDTD. Inset: target image. (c) OM image of the fabricated metasurface. (d) SEM image of the fabricated metasurface. Scale bar in panels c and d: 15 µm. (e) Measured intensity distribution at 26 µm above the metasurface.

We realize an integrated meta-hologram experimentally to demonstrate the versatility of the platform of metasurfaces on silicon photonics. We design a meta-hologram with a size of 40 µm by 40 µm to project an image of the letter “ψ” at 20 µm above the metasurface. The required phase profile, as shown in Figure 5a is found by the Gerchberg–Saxton algorithm [41]. We impart the required phase profile using the phase library in Figure 1e. We perform the full-wave 3D FDTD simulation to verify the design. Figure 5b shows that a simulated holographic image of the letter “ψ” is constructed at 20 µm above the meta-hologram. We use the same process shown in Figure 3a to fabricate the device. Figure 5c and d show the optical microscope (OM) and SEM images of the fabricated meta-hologram. The measured intensity distribution at 26 µm above the metasurface is shown in Figure 5e, which agrees well with the simulation. This demonstration shows the versatility of creating emission with a complicated wavefront beyond the capabilities of conventional grating couplers. The demonstrated on-chip holographic projection can find applications in near-eye display for virtual/augmented reality and single-pixel imaging [11, 42].

## 4 Conclusions

In summary, we have demonstrated versatile shaping of free-space emission, including beam focusing and holographic projection, using a monolithic platform of metasurfaces on silicon photonics. The platform enables versatile wavefront control with a subwavelength resolution beyond the capabilities of conventional grating couplers. Given the meta-atom library, one can realize arbitrary emission phase profiles simply by the arrangement of meta-atoms without resorting to computationally intensive optimization [43, 44]. This allows the realization of large-area integrated metasurfaces. The integrated metasurfaces can also operate with higher-order mode excitation (see Supplementary Material). Our demonstration at the telecommunication wavelength can potentially extend to the visible and ultraviolet regime by constructing the metasurfaces and waveguides with large-bandgap CMOS-compatible materials such as SiN, TiO_2_, HfO_2_, and AlO_x_ [45–49]. In the current demonstration, we only control the emission phase profile. The platform can be extended to control the intensity and phase profiles simultaneously by engineering the nanopillar diameters and positions (see Supplementary Material) [50]. The demonstrated integrated metasurfaces can be added to the existing silicon photonic ecosystem to provide a versatile interface between the free-space radiation and the guided modes. More free-space applications of silicon photonic integrated circuits can be envisioned with the offered extended functionalities.

## Supplementary Material

Supplementary Material Details

## References

[1] Miller S. A., Chang Y. C., Phare C. T. (2020). Large-scale optical phased array using a low-power multi-pass silicon photonic platform. Optica.

[2] Chang Y. C., Shin M. C., Phare C. T., Miller S. A., Shim E., Lipson M. (2021). 2D beam steerer based on metalens on silicon photonics. Opt. Express.

[3] Poulton C. V., Byrd M. J., Russo P. (2019). Long-range LiDAR and free-space data communication with high-performance optical phased arrays. IEEE J. Sel. Top. Quantum Electron..

[4] Xie W., Komljenovic T., Huang J. (2019). Heterogeneous silicon photonics sensing for autonomous cars [invited]. Opt. Express.

[5] Rogers C., Piggott A. Y., Thomson D. J. (2021). A universal 3D imaging sensor on a silicon photonics platform. Nature.

[6] Acoleyen K. V., Bogaerts W., Jágerská J., Thomas N. L., Houdré R., Baets R. (2009). Off-chip beam steering with a one-dimensional optical phased array on silicon-on-insulator. Opt. Lett..

[7] Kwong D., Hosseini A., Covey J. (2014). On-chip silicon optical phased array for two-dimensional beam steering. Opt. Lett..

[8] Rhee H. W., You J. B., Yoon H. (2020). 32 gbps data transmission with 2D beam-steering using a silicon optical phased array. IEEE Photonics Technol. Lett..

[9] Kuo P. C., Kuo S. I., Wang J. W. (2022). Actively steerable integrated optical phased array (OPA) for optical wireless communication (OWC). Optical Fiber Communication Conference.

[10] Meynard B., Martinez C., Fowler D., Molva E. (2020). SiN photonic integrated circuit designed to evaluate its interaction with a hologram for an augmented reality application. Proc. SPIE.

[11] Notaros J., Raval M., Notaros M., Watts M. R. (2019). Integrated-phased-array-based visible-light near-eye holographic projector. Conference on Lasers and Electro-Optics.

[12] Mohanty A., Li Q., Tadayon M. A. (2020). Reconfigurable nanophotonic silicon probes for sub-millisecond deep-brain optical stimulation. Nat. Biomed. Eng..

[13] Shim E., Chen Y., Masmanidis S., Lipson M. (2016). Multisite silicon neural probes with integrated silicon nitride waveguides and gratings for optogenetic applications. Sci. Rep..

[14] Mehta K. K., Bruzewicz C. D., McConnell R., Ram R. J., Sage J. M., Chiaverini J. (2016). Integrated optical addressing of an ion qubit. Nat. Nanotechnol..

[15] Mehta K. K., Zhang C., Malinowski M., Nguyen T. L., Stadler M., Home J. P. (2020). Integrated optical multi-ion quantum logic. Nature.

[16] Giewont K., Hu S., Peng B. (2019). 300-mm monolithic silicon photonics foundry Technology. IEEE J. Sel. Top. Quantum Electron..

[17] Yulaev A., Zhu W., Zhang C. (2019). Metasurface-integrated photonic platform for versatile free-space beam projection with polarization control. ACS Photonics.

[18] Zadka M., Chang Y. C., Mohanty A., Phare C. T., Roberts S. P., Lipson M. (2018). On-chip platform for a phased array with minimal beam divergence and wide field-of-view. Opt. Express.

[19] Kim S., Westly D. A., Roxworthy B. J. (2018). Photonic waveguide to free-space Gaussian beam extreme mode converter. Light Sci. Appl..

[20] Su T., Scott R. P., Djordjevic S. S. (2012). Demonstration of free space coherent optical communication using integrated silicon photonic orbital angular momentum devices. Opt. Express.

[21] Mehta K. K., Ram R. J. (2017). Precise and diffraction-limited waveguide-to-free-space focusing gratings. Sci. Rep..

[22] Vermeulen D., Selvaraja S., Verheyen P. (2010). High-efficiency fiber-to-chip grating couplers realized using an advanced CMOS-compatible silicon-on-insulator platform. Opt. Express.

[23] Cardenas J., Poitras C. B., Luke K., Luo L. W., Morton P. A., Lipson M. (2014). High coupling efficiency etched facet tapers in silicon waveguides. IEEE Photonics Technol. Lett..

[24] Khorasaninejad M., Capasso F. (2017). Metalenses: versatile multifunctional photonic components. Science.

[25] Dorrah A. H., Capasso F. (2022). Tunable structured light with flat optics. Science.

[26] Chen M. K., Wu Y., Feng L. (2021). Principles, functions, and applications of optical meta-lens. Adv. Opt. Mater..

[27] Qiao P., Yang W., Chang-Hasnain C. J. (2018). Recent advances in high-contrast metastructures, metasurfaces, and photonic crystals. Adv. Opt. Photonics.

[28] Choudhury S. M., Wang D., Chaudhuri K. (2018). Material platforms for optical metasurfaces. Nanophotonics.

[29] Arbabi A., Arbabi E., Kamali S. M., Horie Y., Han S., Faraon A. (2016). Miniature optical planar camera based on a wide-angle metasurface doublet corrected for monochromatic aberrations. Nat. Commun..

[30] Khorasaninejad M., Chen W. T., Oh J., Capasso F. (2016). Super-dispersive off-Axis meta-lenses for compact high resolution spectroscopy. Nano Lett..

[31] Li L., Liu Z., Ren X. (2020). Metalens-array–based high-dimensional and multiphoton quantum source. Science.

[32] Ha Y., Guo Y., Pu M. (2020). Minimized two- and four-step varifocal lens based on silicon photonic integrated nanoapertures. Opt. Express.

[33] Ha Y., Guo Y., Pu M. (2021). Monolithic-integrated multiplexed devices based on metasurface-driven guided waves. Adv. Theory Simul..

[34] Ding Y., Chen X., Duan Y. (2022). Metasurface-dressed two-dimensional on-chip waveguide for free-space light field manipulation. ACS Photonics.

[35] Guo X., Ding Y., Chen X., Duan Y., Ni X. (2020). Molding free-space light with guided wave–driven metasurfaces. Sci. Adv..

[36] Li Z., Kim M. H., Wang C. (2017). Controlling propagation and coupling of waveguide modes using phase-gradient metasurfaces. Nat. Nanotechnol..

[37] Fang B., Wang Z., Gao S., Zhu S., Li T. (2021). Manipulating guided wave radiation with integrated geometric metasurface. Nanophotonics.

[38] He T., Meng Y., Liu Z. (2021). Guided mode meta-optics: metasurface-dressed waveguides for arbitrary mode couplers and on-chip OAM emitters with a configurable topological charge. Opt. Express.

[39] Khorasaninejad M., Zhu A. Y., Roques-Carmes C. (2016). Polarization-insensitive metalenses at visible wavelengths. Nano Lett..

[40] Liu V., Fan S. (2012). S4: a free electromagnetic solver for layered periodic structures. Comput. Phys. Commun..

[41] Gerchberg R. W. (1972). Practical algorithm for the determination of phase from image and diffraction plane pictures. Optik.

[42] Fukui T., Kohno Y., Tang R., Nakano Y., Tanemura T. (2021). Single-pixel imaging using multimode fiber and silicon photonic phased array. J. Light. Technol..

[43] Yang K. Y., White A. D., Ashtiani F. (2021). Inverse-designed multi-dimensional silicon photonic transmitters. ..

[44] Shen B., Wang P., Polson R., Menon R. (2014). Integrated metamaterials for efficient and compact free-space-to-waveguide coupling. Opt. Express.

[45] Khorasaninejad M., Chen W. T., Zhu A. Y. (2017). Visible wavelength planar metalenses based on titanium dioxide. IEEE J. Sel. Top. Quantum Electron..

[46] Fan Z. B., Shao Z. K., Xie M. Y. (2018). Silicon nitride metalenses for close-to-one numerical aperture and wide-angle visible imaging. Phys. Rev. Appl..

[47] Zhang C., Divitt S., Fan Q. (2020). Low-loss metasurface optics down to the deep ultraviolet region. Light Sci. Appl..

[48] Lin C., Peñaranda J. S. D., Dendooven J. (2022). UV photonic integrated circuits for far-field structured illumination autofluorescence microscopy. Nat. Commun..

[49] Aslan M. M., Webster N. A., Byard C. L. (2010). Low-loss optical waveguides for the near ultra-violet and visible spectral regions with Al_2_O_3_ thin films from atomic layer deposition. Thin Solid Films.

[50] Lin Y. S., Hsieh P. Y., Fang S. L., Chang Y. C. (2022). Metasurfaces on silicon photonics for simultaneous emission amplitude and phase control. Conference on Lasers and Electro-Optics.

