# Supplementary material for: Integrated metasurfaces on silicon photonics for emission shaping and holographic projection
Source: Nanophotonics. 2022 Oct 20;11(21):4687–95. doi: 10.1515/nanoph-2022-0344 (PMC11501560; doi:10.1515/nanoph-2022-0344)
Supplement: Supplementary file 1 — Supplementary Material Details [file j_nanoph-2022-0344_suppl.pdf]

Supplementary Material for:

Integrated Metasurfaces on Silicon Photonics for Emission Shaping and Holographic Projection

Ping-Yen Hsieh,[1] Shun-Lin Fang, [1] Yu-Siang Lin, [1] Wen-Hsien Huang,[2] Jia-Min Shieh,[1,2] Peichen Yu,[1] and You-Chia Chang[1,]*

[1]Department of Photonics, College of Electrical and Computer Engineering, National Yang Ming Chiao Tung University, Hsinchu 30010, Taiwan

[2]Taiwan Semiconductor Research Institute, Hsinchu 30078, Taiwan

1. Detailed device structure of the metasurface on a silicon photonic waveguide

Figure S1 shows the schematic of a unit cell of the device. We fabricate the device on a silicon-on-insulator (SOI) wafer with a 220 nm top silicon layer and a 3 μm buried oxide layer. Between the metasurface and the silicon waveguide, we introduce an 80 nm SiO_2 layer for controlling the coupling strength. This layer also serves as the hardmask for the waveguide etching. The metasurface is composed of amorphous silicon (a-Si) nanopillars with a height of 1.2 μm. The nanopillars are arranged in a square lattice with a period of 562 nm. The radius of the pillars ranges

from 100 to 194 nm. There is an 80 nm-thick SiO_2 hardmask left on top of the a-Si nanopillar after metasurface etching.

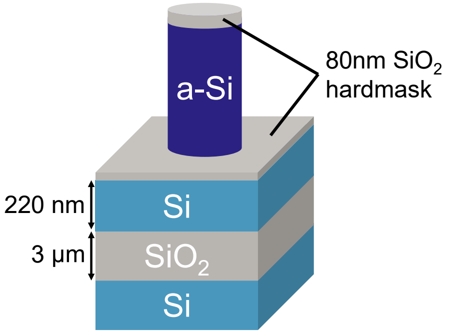

Figure S1. Schematic of a unit cell of the metasurface on the silicon waveguide.

2. Simulation of meta-atom phase library by rigorous coupled-wave analysis (RCWA)

We use RCWA to allow a more efficient search of the design parameters than the more rigorous but computation-intensive 3D finite-difference time-domain (FDTD). Because RCWA only allows plane wave sources, we excite the nanopillars with a total internal reflected (TIR) plane wave source that produces the same evanescent field of the TE_0 slab waveguide mode (Figure S2a). As shown by the simulation model in Figure S2b, we send a plane wave source with $\lambda = 1550$ nm from a bulk silicon. The incident angle θ_i is determined by the condition $n_{Si} k_0 \sin\theta_i = n_{\text{eff}}^{\text{slab}} k_0$, where n_{Si} is the refractive index of the bulk silicon, k_0 is the free-space wave number, and $n_{\text{eff}}^{\text{slab}}$ is the effective refractive index of the TE_0 mode of the waveguide with the nanopillars on top. In order to obtain the effective refractive index $n_{\text{eff}}^{\text{slab}}$ of a waveguide that includes a nanopillar array on its top, we perform a single FDTD simulation shown in Figure S2c. We use the FDTD simulation to find the far-field diffraction angle θ_f and extract $n_{\text{eff}}^{\text{slab}}$ using the grating equation $k_0 \sin\theta_f = n_{\text{eff}}^{\text{slab}} k_0 - \frac{2\pi}{\Lambda}$, where $\Lambda = 562nm$ is the period of the nanopillar array.

The radius of the nanopillar is chosen to be a fixed value of 147 nm in determining $n_{\text{eff}}^{\text{slab}}$. We find the far-field diffraction angle θ_f to be 5.48°, which corresponds to $n_{\text{eff}}^{\text{slab}} = 2.8535$. Once we know $n_{\text{eff}}^{\text{slab}}$, we can calculate corresponding θ_i and use the RCWA simulation to calculate the emission phase library. The results are shown in Figures 1d and 1e in the main text.

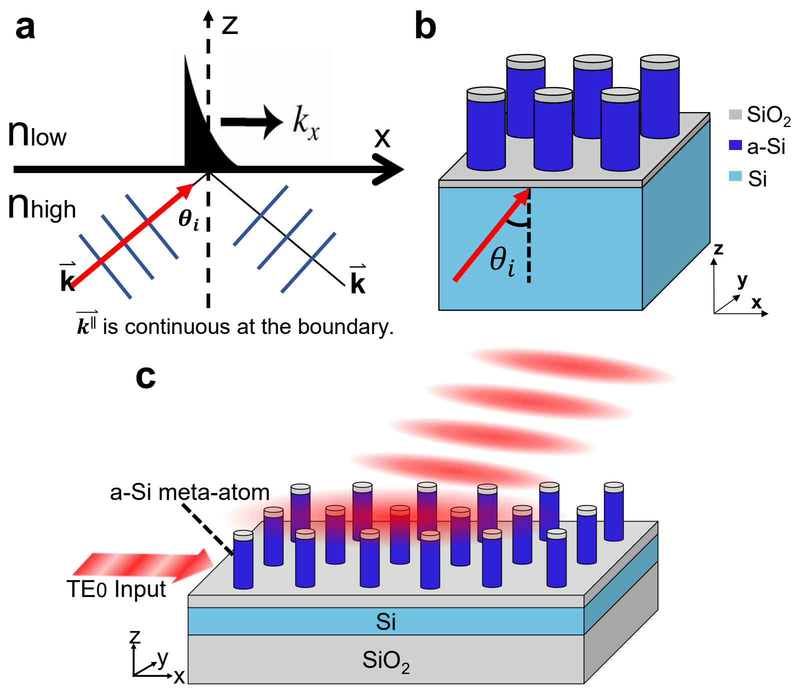

Figure S2. (a) Schematic of the generation an evanescent wave using a plane wave and the total internal reflection. (b) Schematic of the simulation model in RCWA. (c) Schematic of the simulation model in FDTD for determining the effective refractive index $n_{\text{eff}}^{\text{slab}}$. Periodic boundary condition is used in the y direction.

The 562 nm period of the metasurface is chosen to allow easier vertical emission while avoiding the back reflection from the second-order diffraction. Using this period, if all the meta-atoms have identical radii, the structure can be simplified as a grating. The diffraction angle θ_f of this grating is given by the grating equation

$$k_0 \sin\theta_f = \beta - \frac{2\pi}{\Lambda}, \tag{S1}$$

where β is the propagation constant of the waveguide mode. We choose Λ = 562 nm such that the grating diffraction angle θ_f = 5.48°. This is a value close to the vertical emission but has a small angle. This choice is similar to designing a conventional grating coupler for fiber-waveguide coupling [1]. If the grating is designed for the exact vertical emission, the second-order diffraction can phase-match the backward propagation mode and causes strong back reflection. Therefore, a small deviation from the exact vertical emission is commonly used in grating coupler designs.

Although the proposed metasurface can introduce an arbitrary phase shift profile $\phi_{\text{ms}}(x,y)$ by assigning different radii to different meta-atoms, such a choice of the period can keep the profile $\phi_{\text{ms}}(x,y)$ smooth for most vertical emission applications. This can be understood by the following equation. For generating an emission with a phase profile $\phi_{\text{em}}(x,y)$,

$$\begin{aligned}\phi_{\text{em}}(x,y) &= \phi_{\text{ms}}(x,y) + \beta x, \qquad \text{(S2)}\\ &= \phi_{\text{ms}}(x,y) + \left(k_0 \sin\theta_f + \frac{2\pi}{\Lambda}\right)x\\ &= \phi_{\text{ms}}(x,y) + \left(k_0 \sin\theta_f\right)x.\end{aligned}$$

Here we have used the fact that $x = m\Lambda$, where m is an integer. The $(k_0 \sin\theta_f)x$ term creates a tilt to the wavefront. By keeping θ_f small, we can reduce the contribution from the $(k_0 \sin\theta_f)x$ term and keep the profile $\phi_{\text{ms}}(x,y)$ as smooth as possible if the main emission direction is vertical.

3. Simulation of meta-atom phase and amplitude libraries by finite-difference time-domain (FDTD)

We use the same FDTD simulation model described in the previous section (Figure S2c) to calculate the phase and amplitude libraries of the metasurface. We place a monitor above the

meta-atom array and capture the complex emission electric field, including the amplitude and the phase. The procedure is repeated as we sweep through different radii. These results allow us to construct the phase and amplitude libraries.

A phase library provides a look-up table between the nanopillar radius and the phase shift, which is shown in Figure 1e of the main text. To characterize the emission amplitude of the meta-atom, we use a quantity called the leakage parameter [2]. Leakage parameter characterizes the fraction of power that leaks from the waveguide per unit length, as defined by

$$-\frac{dP_{wg}}{dx} = 2[\alpha_{up} + \alpha_{down}]P_{wg}, \tag{S3}$$

where P_{wg} is the power remaining in the waveguide. α_{up} and α_{down} are the leakage parameters for the upward and downward emission, respectively. In the FDTD simulation, we extract the leakage parameters by monitoring the power leaking to the free space and the power remaining in the waveguide. Figure S3 shows the leakage parameter α_{up} as a function of the nanopillar radius, which represents the amplitude library of the metasurface.

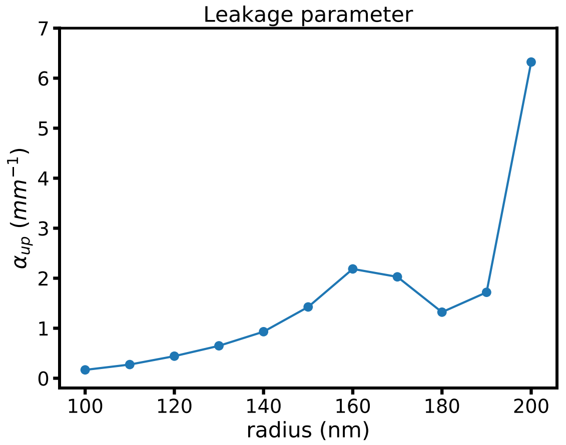

Figure S3. Amplitude library of the metasurface obtained by the FDTD simulation, which shows the leakage parameter α_{up} as a function of the nanopillar radius.

4. Modal analysis of the nanopillar

Each Si nanopillar can be viewed as a truncated cylindrical waveguide [3]. Here we predict the phase shift associated with the eigenmode when the light passes through the Si nanopillar. We use the eigenmode solver (MODE solutions, Lumerical Inc.) to solve the fundamental eigenmode of a silicon nanopillar array arranged in a square lattice with a period = 562nm. We calculate the effective refractive index $n_{\text{eff}}^{\text{pillar}}(r)$ for different nanopillar radius r. The phase shift after passing through the nanopillar $\phi(r) = k_0 n_{\text{eff}}^{\text{pillar}}(r)h$, where k_0 is the free-space wave number, and h is the height of the nanopillar. The result is shown in Figure 1e in the main text.

5. Focusing efficiency calculation

The focusing efficiency is defined as

$$\text{Focusing efficiency} = \frac{\text{Power that reaches the focused spot}}{\text{Input power that enters the waveguide}}. \quad \text{(S4)}$$

We calculate the power that reaches the focused spot by integrating the intensity over a rectangular area on the focal plane. The widths of the rectangular area are chosen to be 6 times the full-width at half-maximum (FWHM) spot sizes in the x and y directions.

6. Strehl ratio calculation

We quantify the focusing quality by the Strehl ratio. We calculate the ideal intensity distribution by using FDTD to simulate the uniform emission from a rectangular aperture of 60 μm × 20 μm with the ideal hyperbolic phase profile given by $\phi(x, y) = -k_0\left(\sqrt{x^2 + y^2 + F^2} - F\right)$. We obtain the ideal intensity distribution on the focal plane $I_{\text{ideal}}(x, y)$, which follows well the sinc^2 function. To calculate the Strehl ratio of the measured data, we capture the intensity

distribution on the focal plane $I_{\text{measure}}(x, y)$. Both $I_{\text{ideal}}(x, y)$ and $I_{\text{measure}}(x, y)$ are normalized to their integrated power, where the integration is taken over a rectangular area with widths of 6 times the FWHM spot sizes in the x and y directions. The ratio between the peak intensities of $I_{\text{measure}}(x, y)$ and $I_{\text{ideal}}(x, y)$ gives the Strehl ratio. A Strehl ratio higher than 0.8 is conventionally considered diffraction-limited.

7. Simulated and measured intensity cross-sections along the y axis

In the main text, we show the simulated and measured intensity cross-sections along the x axis in Figures 2f, 2g, 4d, and 4e. Here we show the results along the y axis. Figures S4a and S4b show the FDTD-simulated intensity cross-sections at the focal plane along the y direction for the two excitation directions. The FDTD-simulated FWHM spot sizes are 8.2 μm and 8.1 μm in Figure S4a and S4b, respectively. For comparison, we also plot the ideal intensity cross-sections produced by the uniform emission from a rectangular aperture of 60 μm × 20 μm with ideal hyperbolic phase profiles. Figures S4c and S4d show the measured intensity cross-sections at the focal plane along the y direction. The measured FWHM spot sizes are 6.5 μm and 5.8 μm in Figure S4c and S4d, respectively.

We notice that the measured FWHM spot sizes along the y direction are smaller than the FDTD-simulated sizes, as shown in Figure S4c and S4d. However, the measured profiles show shoulder-like distortions. These results show that the FWHM spot size does not fully characterize the performance of the focusing performance. Strehl ratio is a better indicator because it is associated with the integrated power over an area. However, the information of the entire intensity distribution $I(x, y)$ is needed to show all the details of the focusing performance.

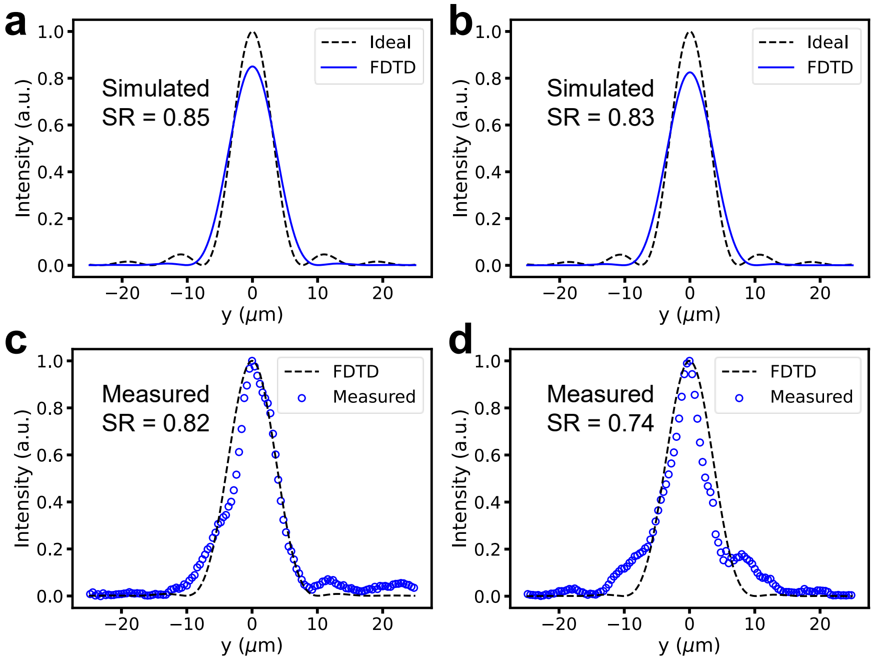

Figure S4. The simulated and measured intensity cross-sections along the y direction. (a,b) Cross-sections of the FDTD-simulated intensity distributions at the focal plane along the y direction when the metasurface is excited by the +x (panel a) and -x (panel b) propagating guided modes. For comparison, we also plot the ideal intensity distributions produced by the uniform emission from a rectangular aperture with ideal hyperbolic phase profiles. The intensity is normalized to the integrated power. (c,d) Cross-sections of the measured and FDTD-simulated intensity distributions at the focal plane along the y direction when the metasurface is excited by the +x (panel c) and -x (panel d) propagating guided modes. The data is normalized to the peak intensity. SR: Strehl ratio.

8. Excitation with the TE_1 mode and the TM_0 mode

Although our metasurface is designed for TE_0 mode excitation, we have performed the 3D FDTD simulation of the metasurface when it is excited with TE_1 and TM_0 modes. The schematic is shown in Figure S5. When the input excitation is in the TE_1 mode (see Figure S6a), the

metasurface produces two focused spots, as shown in Figure S6b-d. To explain the results, we can express the phase profile $\phi_{\mathrm{em}}(x,y)$ emitted to the free space as

$$\phi_{\mathrm{em}}(x,y) = \phi_{\mathrm{ms}}(x,y) + \beta x + \phi_{\mathrm{mode}}(y), \tag{S5}$$

where $\phi_{\mathrm{ms}}(x,y)$ is the abrupt phase shift created by the metasurface, and $\phi_{\mathrm{mode}}(y)$ is the phase profile of the input mode.

We simulate the effective refractive indices n_{eff} of different modes by the eigenmode solver. The n_{eff} of the TE_1 mode in the 22 μm-wide silicon waveguide equals 2.8433, which is very close to the n_{eff} of the TE_0 mode, which equals 2.8439. Therefore, although this metasurface is designed for the TE_0 mode, the emission phase profile using the TE_1 mode is still well-controlled because the βx term remains accurate. The metasurface creates two focused spots because the TE_1 mode is anti-symmetric in the y direction with a node at the center. The TE_1 mode has a π phase shift between the +y and –y portion of the mode, which is inherited by the emission phase profile through the $\phi_{\mathrm{mode}}(y)$ term in Eq. (S5).

When the metasurface is excited by the TM_0 mode (see Figure S7a), the emission intensity distribution is shown in Figure S7b and c. The emission is not well-controlled because the n_{eff} of the TM_0 mode is 1.9819, which is very different from the value of the TE_0 mode. Also, the electric field direction is different from what we use to calculate the meta-atom library (see Figure 1e of the main text). Therefore, for a metasurface designed for the TE_0 mode, the emission phase profile is not well-controlled if we excite it with the TM_0 mode.

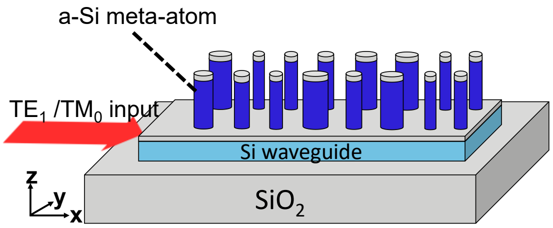

Figure S5. Schematic of the simulation model. We use the TE_1 and TM_0 modes as the input source.

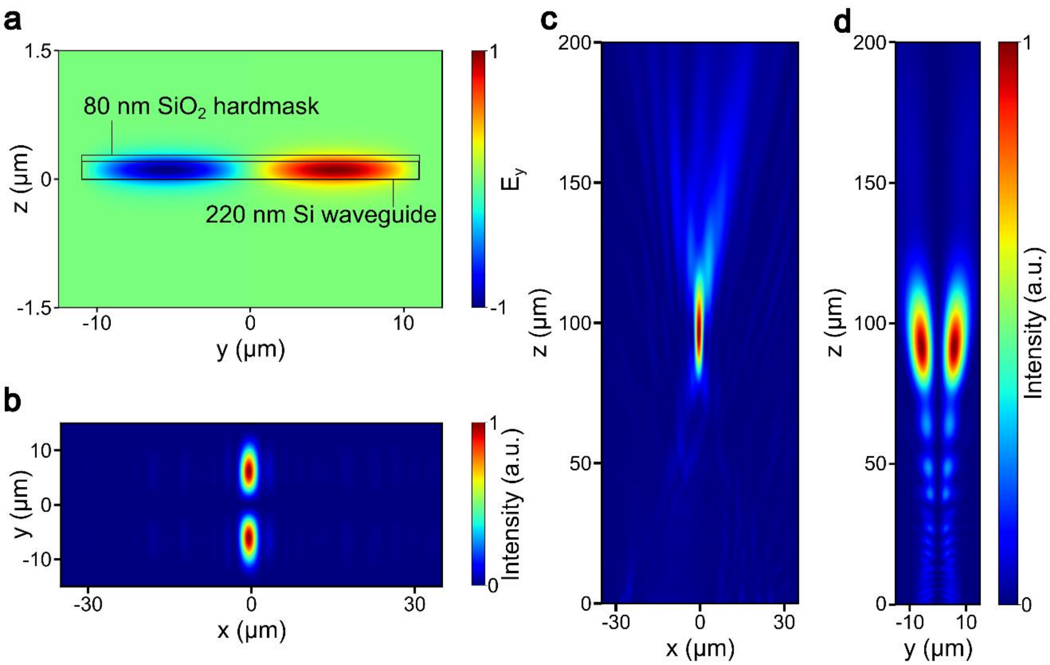

Figure S6. Metasurface emission under TE_1 excitation. (a) E_y profile of the TE_1 mode. (b) Intensity distribution on the focal plane at z = 97 μm. (c) Intensity distribution on the xz plane. (d) Intensity distribution on the yz plane.

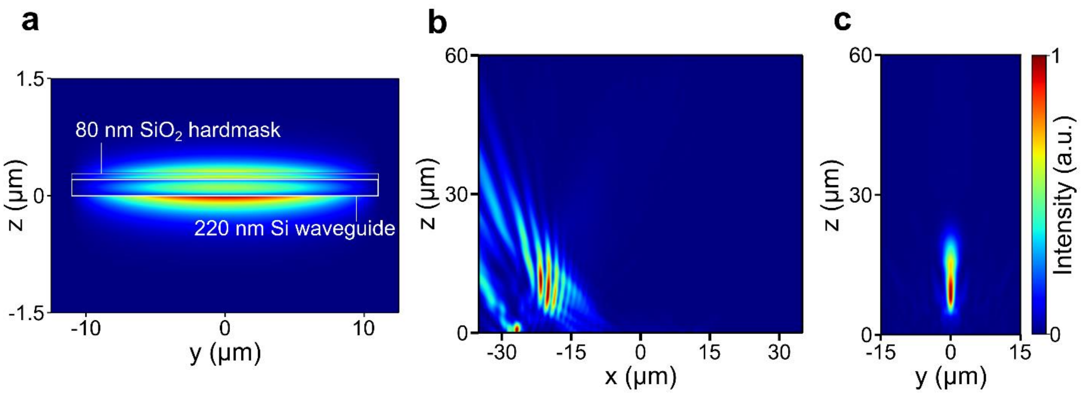

Figure S7. Metasurface emission under TM_0 excitation. (a) E_z profile of TM_0 mode. (b) Intensity distribution on the xz plane. (c) Intensity distribution on the yz plane.

9. Characterization of the waveguide propagation loss

We have characterized the propagation loss of 450 nm-wide silicon waveguides fabricated with our process. Figure S8 shows the measured optical loss for different waveguide lengths, from which we extract the propagation loss to be ~12 dB/cm. This value is higher than the typical propagation loss of 1~2 dB/cm reported in silicon photonic foundries [4]. The propagation loss is introduced by the roughness of the SiO_2 hard mask, which is fabricated by the electron-beam evaporation and lift-off process. One can potentially improve the process by making a higher-quality SiO_2 hard mask with plasma etching. For example, in Reference [5], a high-quality SiO_2 hard mask is fabricated using plasma-enhanced chemical vapor deposition (PECVD) and inductively-coupled plasma reactive-ion etching (ICP-RIE). This hard mask is part of the processes the authors use to achieve ultra-low-loss SiN waveguides with a propagation loss of 0.8 dB/m and SiN microrings with a quality factor of 37 million.

The etching of the amorphous silicon nanopillars is repeatable because the 80 nm SiO_2 layer serves as the etching stop. This SiO_2 layer also serves as the hard mask that defines the waveguides. We calibrate the etching rate carefully. To make sure the silicon layer of the SOI

wafer is fully etched, we intentionally introduce some over-etching into the buried oxide layer. We have performed simulations with the eigenmode solver to ensure the over-etching does not affect the device performance. With 30 nm over-etching, the effective refractive indices n_{eff} of the 22 μm- and 42 μm-wide silicon waveguides change by less than 0.03 %.

We fabricate the whole chip using the process described in the main text. We use grating couplers to couple light from a fiber to the chip. The distances from the two input grating couplers to the 60 μm×20 μm metasurface region (shown in Figure 4 of the main text) are 450 μm and 490 μm, respectively. For the 40 μm×40 μm meta-hologram device (shown in Figure 5 of the main text), the distance from the input grating coupler to the meta-hologram region is 2100 μm.

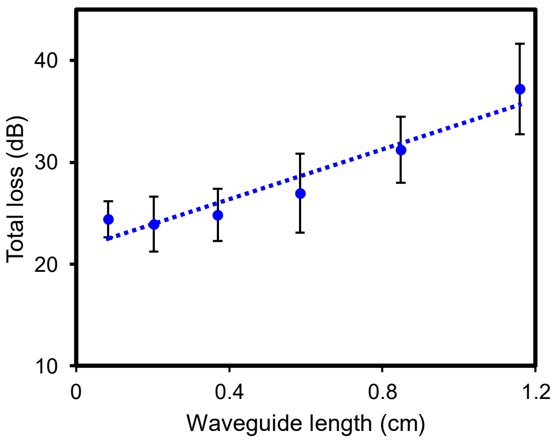

Figure S8. Optical loss measurement with different waveguide lengths. The extracted waveguide propagation loss is 12 dB/cm for 450 nm-wide waveguides.

10. Depth of focus of the measurement setup

In our measurement setup, we move the microscope with a motorized stage to capture a series of images plane by plane along the z axis. The images are broadened because of the finite depth of focus. Here we use a high NA objective (Mitutoyo M Plan APO NIR HR 50X NA 0.65) to minimize the broadening. The depth of focus is given by

$$\text{Depth of focus} = 0.5\frac{\lambda}{NA^2} = 1.83\ \mu\text{m}. \quad \text{(S6)}$$

Because this depth of focus of the microscope is much smaller than the required sectioning resolution to map the intensity distributions emitted from our metasurface (see Figures 4b and 4c of the main text), we do not observe noticeable broadening. This sectioning measurement technique is widely used in the literature to map the focusing profiles of metalenses [6].

11. Control of the intensity profile

As the silicon nanopillars couple light to the free space, the light guided in the waveguide decreases. As a result, the emission intensity profile gradually decreases along the propagation direction. In our work, we choose a weak perturbation and a short metasurface region to keep the decrease of intensity profile small. Consider the metasurface designed for switchable beam focusing. After passing through the 60 μm-long metasurface region, 65 % of power remains in the waveguide. Please see Section 10 for the detailed analysis of the energy flow. Our current approach can work well in applications that only require extracting a small part of the energy or those that can allow gradually-decreasing intensity profiles.

To fully handle the intensity profile, we can extend our approach. Using the same platform of metasurfaces on silicon photonics but with a different design methodology, we can use the nanopillar diameter and position to control the intensity and phase independently, as shown in [7].

12. Analysis of the energy flow from the metasurface

We have analyzed the energy flow from the integrated metasurface designed for switchable beam focusing by 3D FDTD, as summarized by Table S1. Some energy flow goes downward and

underneath the waveguide. The sum of the energy flow is 98% for both +x and –x incident wave excitation directions. The missing 2% is attributed to the numerical error of the FDTD simulation.

When the metasurface is excited by the +x propagating guided mode, 14 % of the energy flow goes upward. 78% of the upward emitting energy flow reaches the focused spot, and therefore the focusing efficiency is 14 % × 78 % = 11 %. When the metasurface is excited by the –x propagating guided mode, 17 % of the energy flow goes upward. 82% of the upward emitting energy flow reaches the focused spot; therefore, the focusing efficiency is 17 % × 82 % = 14 %. Detailed information on calculating the focusing efficiency is given in Section 5 of the supplementary material.

Table S1. Summary of the energy flow from the metasurface. For the Upward and Downward cases, the energy flow emits to the free space. For the Transmission and Back Reflection cases, the energy flow remains in the waveguide.

Incident wave excitation direction	Upward	Downward	Transmission	Back reflection
+x	14%	19%	65%	0%
–x	17%	26%	55%	0%

13. Analysis of the robustness of the metasurfaces

Here we provide a direct comparison of the robustness between the silicon nanopillars and the Au/SiO_2/Au sandwich structure used in Ref. [8]. Figure S9 (a) shows the phase library of the silicon nanopillars. The slope is similar across the library. In the most sensitive region, the slope indicates 0.155π phase error per 10 nm diameter difference. Figure S9 (b) shows the phase library of the Au/SiO_2/Au sandwich structure extracted from the data in Ref. [8]. Here the meta-atom

length in the x direction is fixed at 105 nm, and the length in the y direction (Ly) varies to control the phase. One can observe a sharper 1.1π phase jump when Ly changes from 250 nm to 300 nm. The phase jump is attributed to the electric and magnetic dipole resonances, which can also be observed in other similar systems [9]. The maximum slope corresponds to −0.359π phase error per 10 nm length difference. Comparing the maximum slopes of the two phase libraries, the dimension sensitivity of the silicon nanopillars is 2.3 times more robust than the Au/SiO_2/Au sandwich structure.

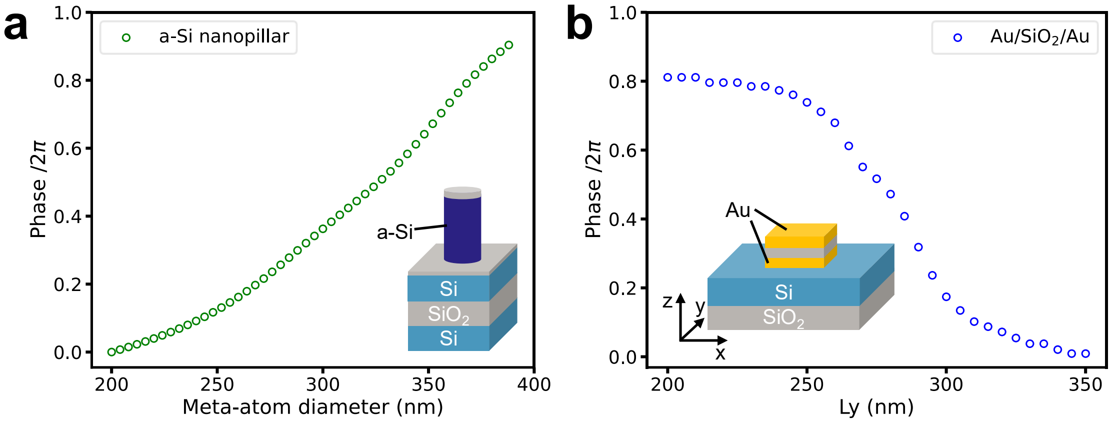

Figure S9. Comparison of the phase libraries of different structures. (a) Phase library of amorphous silicon nanopillars obtained by RCWA. The inset shows the schematic of a meta-atom. (b) Phase library of Au/SiO_2/Au sandwich structure. The raw data is extracted from the colormap in Figure 2B of Ref. [8]. The inset shows the schematic of a meta-atom.

To demonstrate the robustness of the silicon nanopillar metasurface, we perform 3D FDTD simulation of the metasurface with dimension offsets. As shown in Figure S10, even when all the meta-atom diameters are offset by ±30 nm, the metasurface can still produce a tightly-focused spot. The performances of the offset metasurfaces are summarized in Table S2. The diameter offset of ±30 nm only introduces minor degradation of the Strehl ratio. The focusing efficiency slightly increases as the diameter increases due to enhanced out-coupling. These simulation results show

that the silicon nanopillar metasurface is tolerant to overall dimension offset, which can be caused by dose variation in lithography and parameter shift in etching.

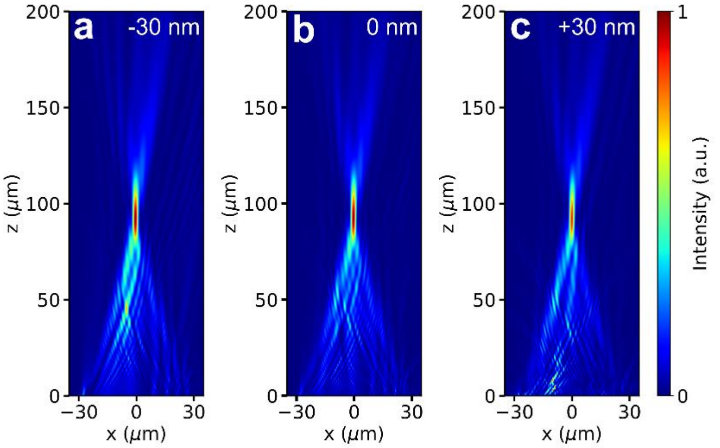

Figure S10. Simulated xz intensity cross sections in the free space when the meta-atoms are offset by (a) –30 nm, (b) 0 nm, (d) +30 nm in diameter.

Table S2. Simulated performances of metasurfaces with different meta-atom diameter offsets.

Diameter offset (nm)	FWHM spot size in x direction (μm)	FWHM spot size in y direction (μm)	Focusing efficiency	Strehl ratio
–30	2.4	8.4	10%	0.81
0	2.3	8.2	11%	0.85
30	2.3	8.1	12%	0.81

The silicon nanopillars support Fabry-Pérot resonance, but the Fabry-Pérot resonance is a minor effect here. Figure S11 (a) and (b) show the normalized emission intensity as a function of meta-atom height. We can observe weak periodic oscillation of the emission intensity, where the period is consistent with the theoretical value of $\lambda/\left(2n_{\mathrm{eff}}^{\mathrm{pillar}}\right) = 477$ nm, where $n_{\mathrm{eff}}^{\mathrm{pillar}}$ is the effective index of the nanopillar. The low visibility of the oscillation indicates that the end facets of the silicon nanopillar do not have sufficient reflections to form a well-confined Fabry-Pérot cavity. Another piece of evidence is the comparison between the phase responses obtained by FDTD and

mode prediction, as shown in Figure S11c. The former takes into consideration the multiple-reflection Fabry-Pérot effect, while the latter calculates the single-pass phase shift $\phi(r) = k_0 n_{\mathrm{eff}}^{\mathrm{pillar}}(r) h$ by treating the nanopillar as a truncated cylindrical waveguide. Here k_0 is the free-space wave number, and h is the height of the nanopillar. The agreement between the two phase responses indicates that the Fabry-Pérot effect only plays a minor role.

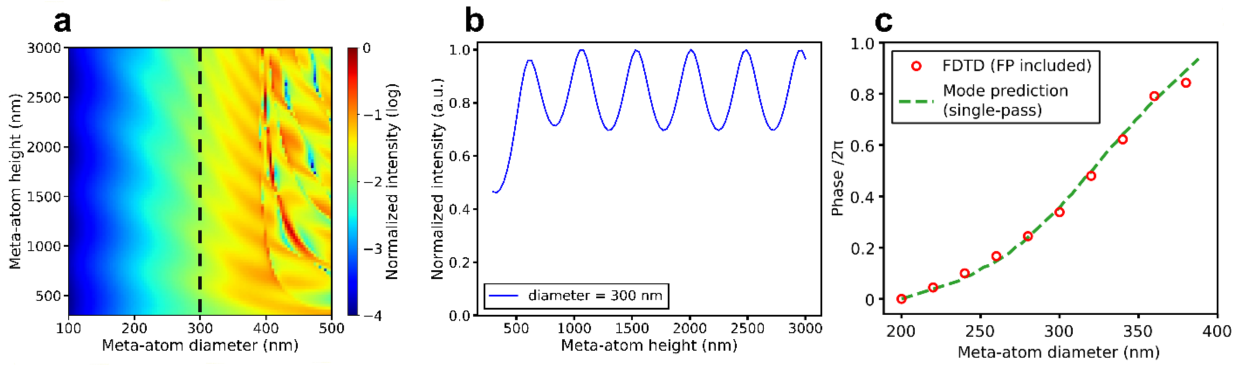

Figure S11. (a) Simulated normalized emission intensity as a function of meta-atom diameter and height obtained by RCWA. (b) Simulated normalized emission intensity when the height changes from 300 nm to 3000 nm. The diameter is fixed at 300 nm (black dashed line in (a)). (c) Phase library comparison between FDTD and mode prediction. FP: Fabry-Pérot

REFERENCES

[1] D. Vermeulen, S. Selvaraja, P. Verheyen, et al., "High-efficiency fiber-to-chip grating couplers realized using an advanced CMOS-compatible Silicon-On-Insulator platform," *Opt. Express* vol. 18, no.17, pp. 18278-18283, 2010.

[2] R. Waldhäusl, B. Schnabel, P. Dannberg, E. B. Kley, A. Bräuer, W. Karthe, “Efficient Coupling into Polymer Waveguides by Gratings,” *Appl. Opt.* vol. *36*, no. 36, pp. 9383, 1997.

[3] M. Khorasaninejad, A. Y. Zhu, C. Roques-Carmes, W. T. Chen, J. Oh, I. Mishra, R. C. Devlin, F. Capasso, "Polarization-Insensitive Metalenses at Visible Wavelengths," *Nano Lett.* vol.16, no. 11, pp. 7229–7234, 2016.

[4] P. P. Absil, P. D. Heyn, H. Chen, et al., "Imec iSiPP25G silicon photonics: a robust CMOS-based photonics technology platform,“ *Silicon Photonics X. SPIE*, vol. 9367, pp. 166-171, 2015.

[5] X. Ji, F. A. S. Barbosa, S. P. Roberts, et al., "Ultra-low-loss on-chip resonators with sub-milliwatt parametric oscillation threshold,“ *Optica*, vol. 4, pp. 619-624, 2017.

[6] M. Khorasaninejad, W. T. Chen, R. C. Devlin, et al., "Metalenses at visible wavelengths: Diffraction-limited focusing and subwavelength resolution imaging," *Science*, vol. 352, no. 6290, pp. 1190–1194, 2016.

[7] Y. S. Lin, P. Y. Hsieh, S. L. Fang, and Y. C. Chang, "Metasurfaces on Silicon Photonics for Simultaneous Emission Amplitude and Phase Control," in *Conference on Lasers and Electro-Optics,* San Jose, CA, 2022*,* p. FF2D.8.

[8] X. Guo, Y. Ding, X. Chen, Y. Duan, and X. Ni, "Molding free-space light with guided wave–driven metasurfaces," *Sci. Adv.* vol. 6, eabb4142 (2020).

[9] K. E. Chong, L. Wang, I. Staude, A. R. James, J. Dominguez, S. Liu, G. S. Subramania, M. Decker, D. N. Neshev, I. Brener, and Y. S. Kivshar, "Efficient Polarization-Insensitive Complex Wavefront Control Using Huygens' Metasurfaces Based on Dielectric Resonant Meta-atoms," *ACS Photonics* vol. 3, pp. 514–519, 2016.
